# Comment on “Exposure to Road Traffic Noise and Behavioral Problems in 7-Year-Old Children: A Cohort Study”

**DOI:** 10.1289/ehp.1510540

**Published:** 2016-02-01

**Authors:** Victor Lezama, Luis Chauca, Maritza Marchena, Daniel Duran

**Affiliations:** 1 Universidad Privada Antenor Orrego, Trujillo, Perú; 2 Instituto Regional de Enfermedades Neoplásicas del Norte, Trujillo, Perú; 3 Hospital Regional Docente de Trujillo, Trujillo, Perú

We were interested to read the article by Hjortebjerg et al. on the relationship between road traffic noise and behavioral problems in children. We were especially interested in the variables considered in this study, because we believe it is insufficient to assess the influence of the noise environment on behavioral disorders of children without also factoring in the impact of parents’ mental and neurological health.

Hjortebjerg et al. mentioned that environmental factors such as noise can cause anxiety and mood swings in adults, and an earlier study published in *EHP* (Power et al. 2011) concluded that environmental noise was associated with cognitive problems in adults. This is relevant because parents are primary influences on the personality and behavior of their children (Méndez-Venegas and Maya-del Moral 2011; Ortiz et al. 2011; Morales et al. 2002). Thus, it is unclear whether variation in children’s behavior is really caused by ambient noise or whether it is a result of disorders in their parents, who themselves may have been influenced by noise exposure. To study the relationship between environmental noise and behavioral problems in children, it is necessary to first rule out problems in parents that may affect their children’s behavior.
